# Remotely Monitored Home-Based Neuromodulation With Transcranial Alternating Current Stimulation (tACS) for Mal de Débarquement Syndrome

**DOI:** 10.3389/fneur.2021.755645

**Published:** 2021-12-09

**Authors:** Yoon-Hee Cha, Jeff Riley, Diamond Gleghorn, Benjamin Doudican

**Affiliations:** ^1^Department of Neurology, University of Minnesota, Minneapolis, MN, United States; ^2^Laureate Institute for Brain Research, Tulsa, OK, United States; ^3^Physician Assistant Studies Graduate Program, Missouri State University, Springfield, MO, United States; ^4^College of Osteopathic Medicine, Oklahoma State University, Tulsa, OK, United States

**Keywords:** Mal de Débarquement Syndrome, oscillating vertigo, transcranial alternating current stimulation, non-invasive brain stimulation, remote-monitoring

## Abstract

**Objective:** To determine whether remotely-monitored transcranial alternating current stimulation (tACS) may be a viable and safe treatment option for Mal de Débarquement Syndrome (MdDS).

**Background:** Mal de Débarquement Syndrome is a neurotological disorder characterized by persistent oscillating vertigo that is triggered by entrainment to passive oscillatory motion such as occurs during water-based travel. Treatment options for MdDS are limited, variably effective, and can be undone by further travel.

**Design and Methods:** This was a remotely-monitored open-label optional extension phase of a double-blind randomized onsite study of tACS for medically refractory MdDS. The primary goal was to determine safety, feasibility, and blinded participant feedback. The secondary goal was to determine efficacy. Thirteen participants (all women), aged 22–67 years, experiencing a duration of illness of 11–72 months, were a subset of 24 individuals who participated in an on-site study of tACS. They had either not responded to the on-site protocol or had relapsed after travel home. Treatment accessories and a tablet controlled tACS stimulator (Pulvinar XCSITE-100) were mailed to participants. Three teaching sessions were performed via webcam followed by on-going remote monitoring of treatment logs and participants' reports through a daily on-line diary and weekly questionnaires. Treatment continued until an effective protocol was administered for 4 weeks and then tapered over 4 weeks. Participants completed a blinded feedback survey and a debriefing interview at the completion of the entire study.

**Results:** Treatment duration ranged from 4 to 31 weeks followed by a 4-week taper accounting for 578 verified sessions. Of the 13 total participants, seven agreed or agreed strongly in the blinded survey that tACS treatment was beneficial; 2) Twelve were comfortable utilizing tACS on their own; 3) Eleven preferred stimulation above their individual alpha frequency; 4) Side effects were generally mild and typical of tACS. In the debriefing interview completed 2–9 months after the last stimulation, five participants reported doing “great,” with no to minimal symptoms, four reported doing “good,” with moderate symptoms, and four reported no change compared to pre-study baseline.

**Conclusion:** Remotely-monitored tACS may be a safe treatment option for MdDS with the potential for lasting outcomes, increased accessibility, and reduction in travel-related treatment reversal.

## Introduction

Mal de Débarquement Syndrome (MdDS) is a neurotological disorder that occurs after exposure to oscillating motion such as from water, air, or land travel (1, 2). The motion perception of MdDS, typically described as a “rocking,” “bobbing,” or “swaying,” is temporarily suppressed by re-exposure to passive motion, but worsens after the motion stops (3). Persistent MdDS lasts for 1-month or longer (1, 4). Structural brain imaging and vestibular function testing do not explain the persistent oscillating vertigo of MdDS but neuroimaging with fMRI and EEG have shown functional connectivity desynchronizations that correlate with symptom improvement that can be induced with both transcranial magnetic stimulation and transcranial alternating current stimulation (5–12).

The challenging feature of MdDS is that it is induced by travel and is worsened by travel (1, 13). Worsening by travel remains a formidable challenge to treating MdDS accounting at least in part for its intractableness since transportation is a necessary part of modern life. Thus, when patients travel for treatment, they often experience recurrence of symptoms simply because of the travel back home. This is true for treatment with non-invasive brain stimulation and with readaptation of the vestibular-ocular reflex (14–18).

Travel-induced worsening of MdDS symptoms necessitated exploring treatments that could be performed at home for extended periods of time. These included remotely monitored home-based neuromodulation options. Non-invasive brain stimulation methods for MdDS have evolved from repetitive transcranial magnetic stimulation (rTMS), rTMS followed by transcranial direct current stimulation (tDCS), theta burst stimulation (TBS), and transcranial alternating current stimulation (tACS) (14–16, 19). Only tDCS and tACS can be performed by the participant on their own given the portability and relative cost of the devices used for treatment.

Home-based tDCS was tried as an adjunctive treatment after induction treatment with 1 and 10 Hz rTMS over dorsolateral prefrontal cortex (DLPFC) in MdDS. All participants had received real rTMS but were randomized to receive either real or sham maintenance tDCS with the anode over F3 and cathode over F4 (15). A total of 556 sessions were performed by 23 participants with a 100% reporting rate. There were no major issues with safety, specifically no episodes of skin burns. This pilot study indicated that home-based noninvasive brain stimulation (NIBS) appeared feasible with high participant satisfaction (15). The device used in that study, which was started in 2013, had a sham mode but did not have monitoring capabilities, however. Therefore, true compliance could not be assessed. Furthermore, though the individuals randomized to real stimulation did better than those randomized sham stimulation, there was not a clinically significant difference in response rate, necessitating further protocol development. Since reduction in fronto-occipital connectivity induced by DLPFC stimulation correlated with reductions in MdDS severity, the next goal was to determine whether connectivity reductions could be induced more directly with tACS (9, 11).

A recently completed tACS study in 24 individuals with MdDS who had a median age of 57 years (range 22–67 years) and median duration of illness of 18-months (range 6–240 months) employed an “n-of-1” design in which all participants received three experimental protocols of fronto-occipital tACS given in a randomized order (19). The protocols were alpha frequency anti-phase (desynchronizing), alpha frequency in-phase (synchronizing), and gamma frequency (40 Hz) anti-phase active sham. Given that MdDS patients have symptoms that are worsened by travel, the treatment study design had to maintain adequate controls while not explicitly allocating patients to sham treatments that were predicted to not impart any benefit and thus knowingly raise the risk of the participants having exacerbated symptoms after traveling home. The participants were thus treated with the protocol that they themselves assessed as lowering their symptoms the most, even if it were the sham condition, after receiving all protocols during a test session. The protocol that they chose was administered for 10–12 sessions over 3 days.

There were participants, however, who were not sure what the most efficacious protocol was and could have potentially chosen a suboptimal protocol in terms of efficacy. Others felt that they had improved but, sometime after they returned home, the MdDS symptoms returned. Traveling back to the study was not a practical option. Therefore, a new option was created for these participants to try the same or a different tACS protocol in a remotely-monitored program, depending on the circumstance, for a longer period of time.

**Figure 1:**
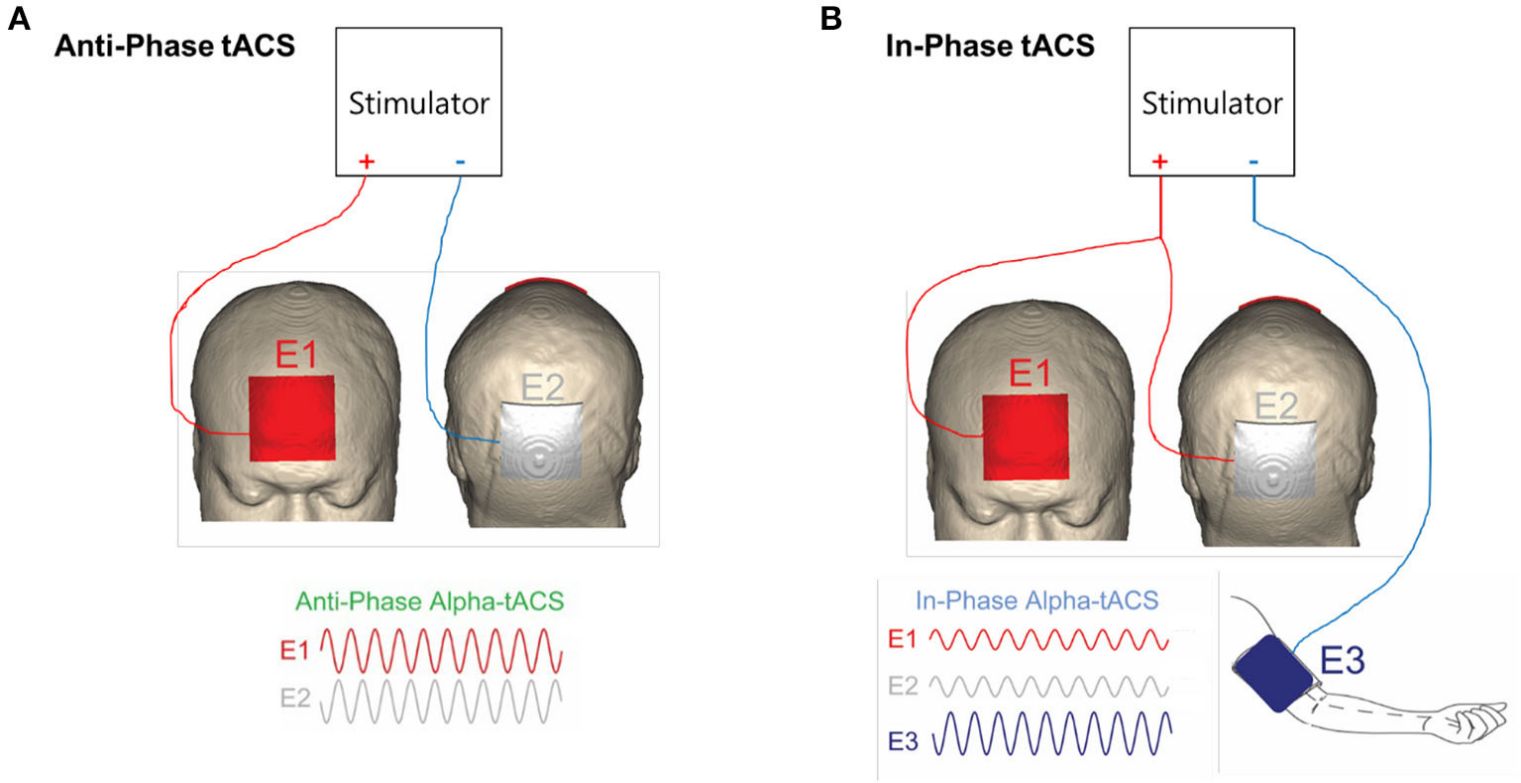
**(A)** Montage for fronto-parietal anti-phase alpha frequency desynchronizing stimulation. **(B)** Montage for fronto-parietal in-phase alpha frequency synchronizing stimulation. Adapted from Ahn et al. (19).

In order to safely provide this therapy, we utilized the Pulvinar XCSITE-100 transcranial electrical current stimulation device in which an accompanying Android tablet controls the stimulator through a Bluetooth connection (https://www.pulvinarneuro.com). A device management tool (TeamViewer.com) allowed the research staff to wirelessly change device settings such as the stimulation frequency (Hz), intensity (mA), and duration (minutes). The investigators could also troubleshoot problems with the participant and use the connection as a safety mechanism to turn off the stimulator if misused. The participants reported side effects for each session through an online personal weblink with these reports being verified against the usage logs reported by the device. Anti-phase vs. in-phase montages were set by whether a current splitting cable was used with a return electrode placed on the arm for the in-phase condition (Figure 1).

The primary aim of this study was to determine the safety, feasibility, and participant feedback of using remotely monitored tACS for MdDS. A secondary aim was to determine whether remotely-monitored user-administered tACS was effective in reducing symptoms of MdDS. If the balance of these features were favorable, NIBS could potentially be used as a primary treatment for neurotological disorders that are at least partially perpetuated by abnormal functional connectivity. It could also be used in an adjunctive manner with other forms of neurotological treatment such as vestibular therapy. The large parameter space for tACS (montage, intensity, frequency, duration), made on-site experimentation prohibitively lengthy and in some contexts (such as during a pandemic in 2021), unsafe. However, if multiple participants could be treated with concurrent protocols managed remotely, experiments could run more efficiently, provide faster feedback, and lead to faster evolution of treatments.

## Methods

All study procedures were approved by the ethics board of Western IRB (www.webirb.com) and were administered consistent with Declaration of Helsinki guidelines. Participants provided written informed consent.

### Recruitment

Participants in an on-site tACS study that involved travel to the study site were given the opportunity to take part in an at-home extension phase of the study. The original group of participants were selected based on meeting inclusion criteria for MdDS, which were consistent with Bárány Society criteria (1) except that their symptoms had to have lasted at least 6-months and they had failed medically available treatments including a trial of a selective serotonin reuptake inhibitor, a benzodiazepine, and physical therapy (19). This was to ensure a favorable risk-to-benefit ratio for trying experimental therapy and to reduce risks of placebo effects.

The on-site study included 24 participants who underwent a 5-day protocol in which baseline assessments including fMRI and EEG were performed on Day 1. On Day 2, the participants received three tACS protocols in randomized order and chose the protocol that they felt most acutely decreased the perception of oscillating vertigo. The protocols were labeled, “1,” “2,” and “3,” with the identity of the protocol blinded to both the participant and the principal investigator. The protocol that the participant decided was the most effective in reducing their vertigo intensity during a 60-min post-stimulation observation period was given over Days 3 through 5. The participants received 3–4 sessions of 20-min of tACS at 2–4 mA each day with a total of 10–12 sessions given over the 3-day period. Day 5 concluded with post-treatment fMRI and EEG (these data will be reported separately). This “n-of-1,” design allowed determination of factors that were important in individual treatment effects and solved an ethical dilemma of explicitly allocating sham stimulation to participants before they traveled home.

The three protocols were as follows: 1) alpha frequency anti-phase, 2) alpha frequency in-phase, 3) gamma frequency anti-phase (active sham) (Figure 1). The order of administration was randomized between participants. Of the 24 participants, 13 chose anti-phase alpha frequency stimulation, 7 chose in-phase alpha frequency stimulation, and 4 chose anti-phase gamma frequency active sham stimulation.

Of the 24 participants, there were 13 who wished to try home therapy either with the same stimulation settings that they had chosen on-site, or to try a new setting, e.g., if, after unblinding, it was revealed that they had chosen the sham stimulation. They could also switch from in-phase to anti-phase or vice versa if they felt that the first protocol that they had tried was not effective. Finally, as we learned during the course of the tACS study, stimulating above the individual alpha frequency (IAF) was more effective than stimulating *at* the IAF. Therefore, most extension phase participants opted to try a slightly higher frequency setting than what had been used in the on-site study.

### Reporting

**Figure 2:**
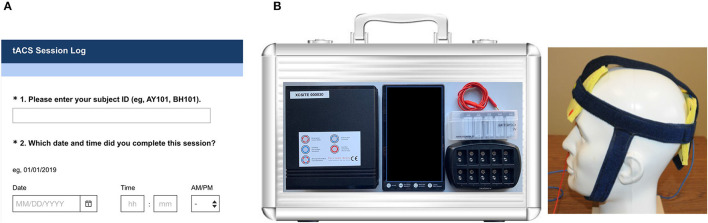
**(A)** Sample screenshot of personalized weblink diary. **(B)** Device case and kit components including the Pulvinar XCSITE 100 stimulator, Android tablet, cables, electrodes, and custom fitted stimulation cap with pre-snapped electrodes.

The participants began reporting symptoms in an online diary 3 weeks before they started treatment in order to determine baseline severity levels of symptoms (Figure 2A). Each diary was entered through a personalized SurveyMonkey® weblink for each participant and reported every weekend. If a set of diaries was not completed by Monday morning, the participants were sent a reminder by email or by phone. Diaries included reports on the Dizziness Handicap Inventory (DHI) (20), the MdDS Balance Rating Scale (MBRS) (15, 16) (Appendix), and the Hospital Anxiety Depression Scale (HADS) (21). Additionally, after each stimulation session, the participant reported their sessions on a SurveyMonkey® link. They reported side effects from a list (Table 1) and rated them from 0 to 10 with 0 being absent and 10 being intolerable.

**Table 1:** Group level distribution percentages of side effects rated at each intensity level for a total of 578 reported stimulation sessions.

**Rating**	**Tingling**	**Itching**	**Redness**	**Headache**	**Tiredness**	**Confusion**	**Nausea**	**Other**
0	25.4	57.8	87.9	68.7	66.8	92.8	87.3	72.7
1	36.9	13.0	8.1	17.5	11.5	5.8	6.9	1.9
2	14.1	9.5	4.0	8.6	10.9	1.2	4.3	4.3
3	8.4	11.6	0.0	3.7	5.7	0.3	1.2	5.6
4	6.6	5.5	0.0	0.9	1.1	0.0	0.3	2.5
5	4.3	2.3	0.0	0.0	2.0	0.0	0.0	6.8
6	2.9	0.0	0.0	0.0	1.1	0.0	0.0	5.0
7	1.4	0.3	0.0	0.3	0.9	0.0	0.0	1.2
8	0.0	0.0	0.0	0.0	0.0	0.0	0.0	0.0
9	0.0	0.0	0.0	0.0	0.0	0.0	0.0	0.0
10	0.0	0.0	0.0	0.3	0.0	0.0	0.0	0.0

*0, absent symptom; 10, intolerable symptom*.

### Device Kits

The participants were mailed a device kit by Week 3 (Figure 2B) that included the Pulvinar XCSITE 100 transcranial electrical current stimulator, an accompanying Android tablet, accessories, and the neoprene headband that had been measured for them during their on-site visit. The Android tablet was pre-programmed with the stimulation app as well as the data management tool TeamViewer for tracking. The participants purchased a single commercial brand of contact lens solution available from a major retailer to use as the conductive medium. Parameters on the app only allowed stimulation to start below a preset impedance level. The stimulation duration, frequency, current level, and impedance threshold could not be changed by the participant.

### Training

Research staff used Skype® or Facetime® to walk through how to use the device and set-up the stimulation sessions with the participants. They performed three sessions with the participant with each subsequent session having the participant perform more and more of the session themselves without prompting. The cap that had been fitted for the participants in the on-site study had electrodes snapped into the headband so that the same location on the head would be stimulated when they donned the cap. They were instructed to wet the entire sponge with saline and to avoid having any saline drip down the face or excessively wet the hair. Any extra saline not in the sponges was to be wiped away. If the hair ended up getting too wet during a set-up session, the participant was instructed to abort that session and try again when their hair was dry. If impedance was too high with just the cap, they could use an elastic head wrap to add pressure to the electrodes.

The participants were instructed to choose a quiet place for the stimulation sessions that would be free from disruptions where they could sit comfortably. They were to perform the stimulations with their eyes closed sitting in a relaxed state. Research staff stayed on-line with the participant until the session ended and were prompted to re-engage when the stimulator provided an auditory alert indicating the end of the session. The research staff then instructed the participant on how to remove the stimulator accessories and keep the components protected until the next session. Once the participants felt comfortable performing the sessions themselves, they were allowed to perform them independently without real-time staff supervision. They were aware that the sessions were being remotely monitored through the device, however, and that online reporting was being followed. Although mild side effects could be reported through the on-line diary, the participants were instructed to report any severe side effects or urgent issues through an email or a phone call to the research staff.

### Stimulation Protocol

All stimulation sessions used a fronto-occipital montage at 2 mA for the anti-phase and 4 mA for the in-phase protocol. The current in the two electrodes for the in-phase protocol were split with a split cable. The return electrode was on the left arm. For the anti-phase protocol, there were two electrodes on the scalp. Stimulation sessions started with 1 session per day for 20-min for 5 days each week. When the study began, the stimulation period was limited to 5 sessions per week for 4 weeks followed by a 4-week taper (4 sessions for 1 week, 3 sessions for 1 week, 2 sessions for 1 week, 1 session for 1 week, then off). However, as we learned that the participants were quite comfortable using the device and were not developing severe side effects, we allowed subsequent participants to use the stimulator for longer periods of time. All participants were tapered for 4-weeks regardless of the total duration of stimulation. There were also periods in which the participant had to take a break because they were traveling. Therefore, there was a wide range of stimulation durations.

Participants reported performing their sessions through a personalized weblink, which included a table for reporting side effects. If the participant felt worse after a tACS session for two consecutive days, the protocol could be adjusted. Otherwise, the participant tried a protocol for at least 2 weeks before they could request a protocol change, e.g., changing the stimulation frequency (Hz). They were maintained on what they considered to be the optimum protocol for 4-weeks before being tapered off.

The number of sessions that the participant reported could be verified in the session files that were reported by the tablet which could be obtained through the device management tool. Once the stimulations were completed and the devices were returned, the participants completed an anonymous participant feedback survey administered through a separate SurveyMonkey® link. They then underwent a debriefing phone interview with the principal investigator (YHC) after all data were collected and the study had concluded. Participants were paid for the weekly diary entries but not for the stimulation sessions.

## Results

Thirteen participants (all women) aged 22–67 years, ranging in duration of illness from 11 to 72 months, participated in the extension phase of the tACS study. There were 23 women and 1 man who participated in the on-site study, which was open to recruitment for both women and men. Triggers for the 13 participants included seven water, five air, and one prolonged residence in a tall swaying tower. All participants had finished high school; two participants had associates degrees, seven had bachelor's degrees, and four had graduate degrees.

### Side Effects

A total of 578 verified sessions were performed by 12 participants with a range of 12–214 sessions and a median of 39 sessions (Table 1). The time stamp on the data logs on the 13th participant could not be extracted to verify against their subjective reports and were thus not counted. Because of the wide range of stimulation session numbers, only the first 40 sessions from the two participants who had performed 90 sessions and 214 sessions were used for group level tabulations after verifying that the spread of side effects reported in the first 40 sessions was similar to the last 40 sessions for these participants. This was to avoid any one participant's experience from overweighting the group level spread of side effects reported.

The main side effects reported were tingling, headaches, itching, and tiredness mostly at a level of 3 or lower (Table 1). There was one report of 10/10 headache by one participant who did not report a score higher than a 2/10 for headache in any other session. Scores as high as 7 were reported by 3 participants who all reported 0's in the same category for other sessions and only on back-to-back days for tingling and phosphenes. In the “Other” category, two participants reported a metallic taste in the mouth and a third reported teeth tingling. One participant noted phosphenes and one a sense of head pulsing. One participant reported heartburn on one occasion. No side effect was severe enough to stop a stimulation session.

### Participant Feedback

The participant feedback survey at the conclusion of the study indicated that most participants found three sessions of training to be sufficient with two participants indicating that more than three sessions would have been helpful (one agreed, one strongly agreed) and six participants disagreeing that more than three sessions would have been helpful (five disagreed, one strongly disagreed) (Table 2). All participants either agreed or agreed strongly that the in-person one-on-one training was sufficient. Overall, nine participants felt comfortable setting up the sessions themselves with four indicating neither agreement nor disagreement. Only one person found that the stimulation sessions were difficult to set-up. Most participants found the online diaries easy to use.

**Table 2:** Anonymous participant feedback survey.

**Statement**	**Strongly agree**	**Agree**	**Neither agree nor disagree**	**Disagree**	**Strongly disagree**	**N/A**
The online diaries were convenient	6	6	1	0	0	0
It was difficult for me to use mobile and online tools.	0	1	0	7	4	1
I felt confident setting up the stimulation sessions.	5	4	4	0	0	0
The stimulation sessions were difficult to set up.	1	0	4	6	2	0
I felt that I had enough in-person one-on-one instruction.	5	8	0	0	0	0
It would have helped to have more in-person one-on-one instruction.	0	1	2	6	4	0
I felt that I was paid enough for my time.	5	4	1	0	1	2
I would have participated without getting paid.	11	1	0	0	1	0
More instruction through Facetime/Skype would have been helpful.	1	1	5	5	1	0
More instruction through Facetime/Skype would have been burdensome.	0	1	8	4	0	0
I felt that the Facetime/Skype sessions were helpful.	8	4	1	0	0	0
Overall, I felt that transcranial electrical stimulation treatment benefited me	2	5	2	2	2	0
How comfortable would you be doing transcranial stimulation on your own without having a physician overseeing your use?	Very comfortable	Somewhat comfortable	Neutral	Somewhat uncomfortable	Very uncomfortable	
	8	5	1	1	0	
How likely are you to participate in a future brain stimulation study?	Very likely	Likely	Not sure	Unlikely	Very Unlikely	
	7	3	3	0	0	

*The questions in the actual survey were presented in randomized order in both a positive and negative direction. They are presented here with like items grouped for clarity*.

Twelve participants indicated that they were “very comfortable,” or “somewhat comfortable,” in managing these sessions without supervision. Two participants had responded to this question twice (thus yielding 15 responses), but only one had responded with both “somewhat comfortable,” and “somewhat uncomfortable,” indicating some ambiguity. Given the small number of participants, there was no demographic factor such as age or education level that predicted whether a participant would need more instruction or supervision than was given with this study design.

In the ongoing feedback with the participants, the main difficulty in stimulation set-up was in knowing how much saline to use on the electrode pads. The participants were sometimes frustrated when the stimulation sessions would not start due to high impedance measures that required restarting the sessions multiple times. This also affected when they could find time in the day to do the sessions which had to be clear of other distractions. Overall, once the participants could determine a good method for maintaining an adequate degree of sponge hydration, they reported a high level of confidence and facility in managing their own treatment.

### Efficacy

In the blinded survey, seven of the 13 participants indicated that they “strongly agreed,” or “agreed,” that tACS was beneficial to them while four “disagreed,” or “strongly disagreed” that it was beneficial and two neither agreed nor disagreed (Table 2). Participants completed weekly reports of the DHI, MBRS, and HADS. Because each participant experienced a different number of treatment weeks, the last 4 weeks of treatment and the 4 weeks of the tapering phase are presented in Tables 3A–D for the 12 participants who had verifiable treatment sessions that could be aligned with the diaries.

**Table 3A:** Dizziness handicap inventory (DHI).

					**Last 4 weeks of stimulation change in DHI**					**4 weeks of taper change in DHI**					
**Participant**	**IAF**	**Final Treatment**	**Relative IAF**	**Pre-stimulation median DHI**	**Stim 1**	**Stim 2**	**Stim 3**	**Stim 4**	**Change (%)**	**Week 1**	**Week 2**	**Week 3**	**Week 4**	**Change (%)**	**Subjective**
1	7.8	In-phase	(+) 0.8–1.1	54	−14	−22	−20	−22	−41	−30	−36	−44	−42	−78	Great
2	8.1	Anti-phase	(+) 0.4–0.7	53	−45	−39	−49	−49	−92	−41	−47	−45	−41	−77	Great
3	9.6	In-phase	(+) 0.4–0.7	13	3	1	−5	−5	−38	−3	−7	−9	−7	−54	Great
4	8.3	In-phase	(+) 0.4–0.7	36	−34	−32	−34	−32	−89	−32	−18	−18	−16	−44	Great
5	8.9	In-phase	(+) 0.8–1.1	46	−2	−2	−10	−20	−43	−10	−16	−12	N/A	−26	Good
6	8.6	Anti-phase	(+) 0.4–0.7	47	−9	−5	−7	1	2	−1	1	−17	−9	−19	None
7	10.4	In-phase	(+) 0.4–0.7	24	−6	−12	−6	−6	−25	−6	−2	−6	−2	−8	Good
8	9.3	Anti-phase	(+) 0.4–0.7	43	−1	5	3	5	12	3	5	1	−3	−7	None
9	7.9	In-phase	(+) 0.4–0.7	82	−2	4	−14	−6	−7	4	4	6	2	2	None
10	10.4	In-phase	0	64	6	6	−4	−2	−3	N/A	20	N/A	N/A	31	None
11	9.6	Anti-phase	(+) 0.4–0.7	48	14	18	18	20	42	16	26	18	16	33	Good
12	8.6	In-phase	(+) 0.4–0.7	31	−3	−3	−3	−3	−10	N/A	N/A	N/A	N/A	N/A	Great

**Table 3B:** Mal de Débarquement balance rating scale (MBRS).

					**Last 4 weeks of stimulation change in MBRS**					**4 weeks of taper change in MBRS**				
**Participant**	**IAF**	**Final Treatment**	**Relative IAF**	**Pre-stimulation median MBRS**	**Stim 1**	**Stim 2**	**Stim 3**	**Stim 4**	**Change (%)**	**Week 1**	**Week 2**	**Week 3**	**Week 4**	**Change (%)**	**Subjective**
1	7.8	In-phase	(+) 0.8–1.1	5	−2	−2	0	0	0	0	−2	−3	−3	−60	Great
3	9.6	In-phase	(+) 0.4–0.7	2	0	1	0	0	0	0	−1	0	−1	−50	Great
2	8.1	Anti-phase	(+) 0.4–0.7	4.5	−2.5	−1.5	−2.5	−2.5	−56	−1.5	−2.5	−1.5	−1.5	−33	Great
5	8.9	In-phase	(+) 0.8–1.1	5	−1	0	0	−2	−40	−2	0	−1	N/A	−20	Good
9	7.9	In-phase	(+) 0.4–0.7	7	−1	−1	−1	−1	−14	0	0	0	−1	−14	None
4	8.3	In-phase	(+) 0.4–0.7	5	−3	−3	−3	−3	−60	−2	0	0	0	0	Great
8	9.3	Anti-phase	(+) 0.4–0.7	6	0	0	0	0	0	0	0	0	0	0	None
6	8.6	Anti-phase	(+) 0.4–0.7	6	0	0	−1	2	33	−1	0	0	0	0	None
7	10.4	In-phase	(+) 0.4–0.7	3.5	−0.5	−0.5	−0.5	−0.5	−14	−0.5	0.5	0.5	0.5	14	Good
10	10.4	In-phase	0	6	2	0	0	0	0	N/A	1	N/A	N/A	17	None
11	9.6	Anti-phase	(+) 0.4–0.7	6	2	2	2	2	33	2	1	2	1	17	Good
12	8.6	In-phase	(+) 0.4–0.7	6	−3	−2	0	−1	−17	N/A	N/A	N/A	N/A	N/A	Great

**Table 3C:** Hospital anxiety depression scale-depression subscale (HADD).

					**Last 4 weeks of stimulation change in HADD**					**4 weeks of taper change in HADD**					
**Participant**	**IAF**	**Final Treatment**	**Relative IAF**	**Pre-stimulation median HADD**	**Stim 1**	**Stim 2**	**Stim 3**	**Stim 4**	**Change (%)**	**Week 1**	**Week 2**	**Week 3**	**Week 4**	**Change (%)**	**Subjective**
5	8.9	In-phase	(+) 0.8–1.1	6	4	−2	1	−4	−67	−1	−2	−5	N/A	−83	Good
1	7.8	In-phase	(+) 0.8–1.1	3	0	0	−1	−2	−67	−2	−2	−2	−2	−67	Great
2	8.1	Anti-phase	(+) 0.4–0.7	9.5	−6.5	−4.5	−6.5	−5.5	−58	−4.5	−8.5	−5.5	−5.5	−58	Great
9	7.9	In-phase	(+) 0.4–0.7	12	−1	−3	−7	−4	−33	−8	−4	−5	−5	−42	None
12	8.6	Anti-phase	(+) 0.4–0.7	10.5	−3.5	−2.5	−5.5	−2.5	−24	−5.5	−3.5	−5.5	−2.5	−24	None
11	9.6	Anti-phase	(+) 0.4–0.7	10.5	−7.5	−5.5	−5.5	−4.5	−43	−3.5	−5.5	−2.5	−1.5	−14	Good
4	8.3	In-phase	(+) 0.4–0.7	3.5	−3.5	−0.5	−3.5	−2.5	−71	−1.5	0.5	2.5	0.5	14	Great
8	9.3	Anti-phase	(+) 0.4–0.7	5	3	4	5	5	100	5	4	5	2	40	None
10	10.4	In-phase	0	9	4	3	−2	−2	−22	N/A	5	N/A	N/A	56	None
7	10.4	In-phase	(+) 0.4–0.7	3	2	−1	3	1	33	1	2	−1	2	67	Good
3	9.6	In-phase	(+) 0.4–0.7	1.5	−0.5	1.5	−0.5	−1.5	−100	−1.5	5.5	1.5	4.5	300	Great
6	8.6	In-phase	(+) 0.4–0.7	7	0	0	0	−1	−14	N/A	N/A	N/A	N/A	N/A	Great

**Table 3D:** Hospital anxiety depression scale-anxiety subscale (HADA).

					**Last 4 weeks of stimulation change in HADA**					**4 weeks of taper change in HADA**					
**Participant**	**IAF**	**Final Treatment**	**Relative IAF**	**Pre-stimulation median HADA**	**Stim 1**	**Stim 2**	**Stim 3**	**Stim 4**	**Change (%)**	**Week 1**	**Week 2**	**Week 3**	**Week 4**	**Change (%)**	**Subjective**
5	8.9	In-phase	(+) 0.8–1.1	4	−3	−4	−3	−4	−100	−3	−4	−4	N/A	−100	Good
2	8.1	Anti-phase	(+) 0.4–0.7	4	−2	−2	−3	−3	−75	−3	−2	−3	−3	−75	Great
4	8.3	In-phase	(+) 0.4–0.7	6.5	−4.5	−4.5	−4.5	−4.5	−69	−4.5	−4.5	−2.5	−3.5	−54	Great
11	9.6	Anti-phase	(+) 0.4–0.7	13.5	−7.5	−4.5	−5.5	−4.5	−33	−7.5	−5.5	−6.5	−6.5	−48	Good
6	8.6	Anti-phase	(+) 0.4–0.7	9.5	−4.5	−6.5	−7.5	−2.5	−26	−8.5	−3.5	−5.5	−4.5	−47	None
9	7.9	In-phase	(+) 0.4–0.7	11	0	1	−4	−3	−27	0	0	−3	−2	−18	None
7	10.4	In-phase	(+) 0.4–0.7	3.5	−1.5	0.5	−0.5	−0.5	−14	−1.5	−1.5	−2.5	−0.5	−14	Good
1	7.8	In-phase	(+) 0.8–1.1	1	−1	0	−1	−1	−100	−1	−1	−1	0	0	Great
10	10.4	In-phase	0	11	1	0	−2	−1	−9	N/A	1	N/A	N/A	9	None
3	9.6	In-phase	(+) 0.4–0.7	1.5	−0.5	−0.5	1.5	−0.5	−33	−1.5	−0.5	−1.5	1.5	100	Great
8	9.3	Anti-phase	(+) 0.4–0.7	5.5	3.5	2.5	4.5	3.5	64	3.5	4.5	5.5	5.5	100	None
12	8.6	In-phase	(+) 0.4–0.7	6	1	1	2	1	17	N/A	N/A	N/A	N/A	N/A	Great

*Participant IDs are anonymously labeled in **(A)** according to greatest change in DHI after the 4 weeks taper. The same IDs are preserved for **(B–D)**. IAF, Individual Alpha Frequency. Column 4 reflects the final treatment frequency used relative to the IAF*.

**Figure 3:**
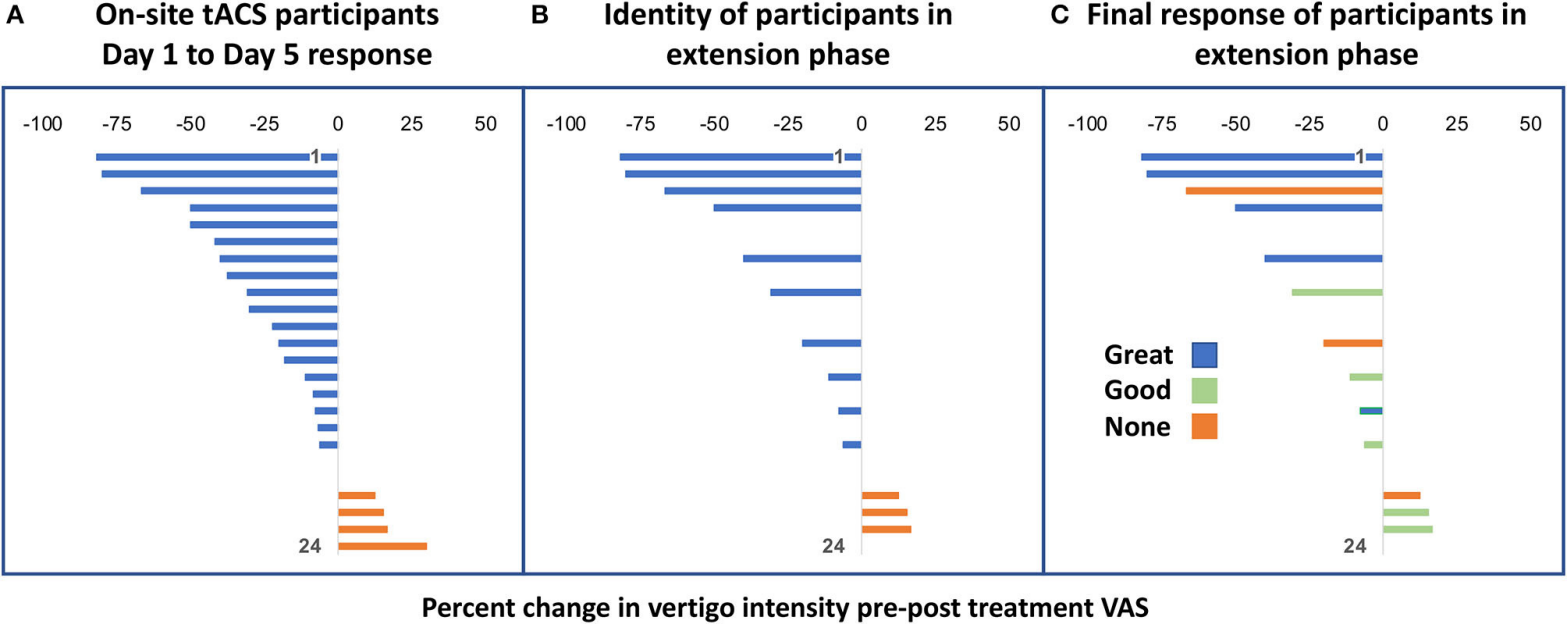
**(A)** Percentage change of oscillating vertigo intensity from Day 1 to Day 5 of on-site tACS treatment of 24 participants. **(B)** Identity of the 13 participants in the extension phase from within the on-site group of 24 participants. **(C)** Final treatment response of the 13 participants as “Great,” “Good,” or “None” shown in relation to their original response level from their on-site participation.

In the debriefing interview at the conclusion of the study, which was about 2–9 months after the final taper, five participants indicated that they were doing “great,” with very minimal symptoms, four participants felt that they were doing, “good,” in which some aspects of their MdDS symptoms were better but not all (e.g., no resolution of rocking but resolution of brain fog); and four participants reported having had no change from baseline. A direct comparison with the blinded survey could not be done due to the survey being anonymized, but the spread of feedback in the open interview was consistent with reports in the blinded survey. Figure 3 shows where the participants in the extension phase study fell in the treatment spectrum of the on-site study and whether they ultimately ended up indicating their response to treatment as “Great,” “Good,” or “None.” Efficacy appeared to correlate best with reductions in the DHI score in terms of whether the participant felt that they had had a response to the treatment (Tables 3A–D).

One of the participants who reported no response subsequently developed true rotational vertigo episodes about 6 months after finishing the taper and was diagnosed with venous and arterial thoracic outlet syndrome. In retrospect, there were some symptoms of this prior to study inclusion. Thus, while no other experimental treatments were undertaken in the interim between the end of the tACS taper and when feedback was obtained, the emergence of other diagnoses, the effect of clinically available medication changes, and lifestyle modifications in the interim could not be ruled out in interpreting longer term efficacy.

## Discussion

This study investigated the feasibility, safety, participant satisfaction, and efficacy in remotely monitored home-based tACS for MdDS. This adds MdDS to the growing list of neurological disorders for which NIBS has been used to treat refractory symptoms (22–24). Because the very large parameter space for tACS requires refinement and tailoring, we focused on whether a home-based stimulation platform that allowed the participant to self-administer the treatment sessions may be safe as well as practical for exploring this parameter space. In order to do this, the platform needed to be controlled remotely, have safety restrictions, and be user-friendly enough to encourage adherence.

### Safety

Side effects were similar to those reported in tDCS and tACS studies and generally did not last past the duration of stimulation sessions (23). This is with the caveat that the majority of human NIBS studies stay within 1–2 mA ranges of stimulation intensity, corresponding to a current density of <0.15 mA/cm2 (25). In this study, the in-phase stimulation was administered at 4 mA split into two electrodes, yielding a maximum current density of 0.02 mA. Rare persistent skin irritations have been reported as well as unmasking hypomania or mania in patients with either unipolar or bipolar disorder in other NIBS studies (26, 27). In the thousands of sessions of tDCS and tACS that have been reported to date, the overall safety profile has been excellent.

The vast majority of home-based transcranial electrical stimulation trials have used tDCS as it has been used more widely and earlier than tACS in laboratory settings. So far, stimulation side effects appear to be very similar between the two modalities, but frequency related phosphenes can be induced by alternating current both by retinal and cortical stimulation in tACS which pose additional challenges in safety and adequate blinding in controlled studies (28, 29).

Side effects in this home-based study were generally mild with the rare severe side effect being inconsistent. All stimulation sessions were completed and there were no reports of skin burns. While there were 13 individuals who completed a very high number of sessions safely, it should be noted that all participants had previously been screened to be in generally good health, had normal structural brain MRIs, had no implanted devices (e.g., pacemakers), and had tolerated in-person treatment sessions with tACS. Risks are not generalizable for individuals who do not pass similar safety screens.

### Monitoring

Despite side effects being uncommon, monitoring is still warranted for potential rare events in NIBS studies (26, 27, 30, 31). The goal for home-based treatment is for research participants and eventually patients to be able to use the simulation devices independently, but safely, and to develop judgment on when to ask for assistance.

The strategy we employed of providing at least three concurrent monitored sessions of tACS and being available for questions by email and phone worked well for our particular cohort of research participants. Despite the majority of the sessions not being monitored in real time, the participants did know that the data that they were entering was being monitored. They were also assessed every couple of weeks to determine whether the protocol they were currently using was efficacious. Although this may have introduced more variability in determining efficacy, it allowed us to test the ability of the research staff to remotely access the tablet that controlled the stimulator box in order to change the protocol. The participant was always aware of when a protocol was being changed.

We used two levels of monitoring. First, the Pulvinar XCSITE-100 device creates a user log that has a timestamp. Remotely accessing the user log allowed the research staff to verify whether the participants had performed the stimulation sessions. They could also determine how many sessions the participants had triggered before they could get a successful session indicating how difficult the participants found setting up the sessions. The main reason for multiple session initiations was the impedance being too high.

Second, the participants were asked to report any side effects in an online diary for each session. This allowed the research staff to cross check participant reports with the simulator output reports. The participants also completed a more intensive questionnaire once a week. The questionnaires could be completed within 30 min with the time to completion recorded. While the intensive data collection created much more research personnel time for tracking, it also served as a reminder to the participant that they were actively in a study despite not being engaged in ongoing live interactions. This helped the study maintain a high adherence rate.

### Participant Feedback

Overall, participants reported high confidence in performing the sessions and using the online tools. All participants felt that they had enough one-on-one instruction. One person felt that more online helped through the webcam would have been helpful while one participant felt that more webcam help would have been burdensome. Most participants were satisfied with the level of monitoring but one participant reported feeling that she was somewhat on her own. This highlights the difficulty in balancing the amount of supervision that participants need for confidence in self-management versus their sense that they are still sufficiently monitored. The adequacy of time was not for technical expertise in performing the sessions, of which all participants became quite competent very quickly. Rather, it was how much interaction with the research staff participants needed to feel that they were receiving adequate attention throughout the course of their treatment. While some individuals may welcome the autonomy of self-treatment that NIBS provide, other individuals may feel that they have missed out on an important part of the therapeutic effect by not meeting with the treatment provider in person.

### Feasibility

Feasibility of managing a remote treatment study required adequate staffing for monitoring reports, funding for mailing the device kits back and forth, and having the ability to troubleshoot technical issues in real time.

From a study management standpoint, the main issues the investigators faced involved keeping track of the large amount of incoming data. The participants were instructed to contact the study coordinators immediately if they had any serious or concerning side effects but less urgent issues were reported in the diaries. When participants requested a change of protocol, an assessment had to be made about the role of contributing factors such as recent travel, sleep deprivation, weather changes, work, or stress in the treatment response. Other monitoring work involved following up on incomplete questionnaires, answering participant questions, and trouble-shooting electronics issues. Managing these issues required significant personnel time.

The participants were mailed the kits and were provided with paid postage to return the kits. Over time, postage costs add up. Because this was a study, the participants were paid to complete the diaries but were not paid for individual sessions which were tailored around their individual treatment responses. A few devices had to be switched out for repairs in the middle of the sessions. All devices were returned at the end of the study. If the devices had not been returned, this would have amounted to a large monetary loss to the study. However, the data management tool allowed the device to be made unusable if not returned and was fortunately not needed for this purpose.

### Efficacy

This home-based treatment program allowed the participants to try different protocols in terms of frequency of stimulation relative to their IAF with almost all participants eventually treated at a frequency above their IAF. The remote nature of the study allowed more time to tailor these treatment parameters. In the blinded survey at the conclusion of the study, seven out of 13 participants indicated benefit from the additional exposure to tACS which closely matched the five out of 13 participants who noted doing “great” and three participants who indicated doing “good,” in the open interview. Considering that the participants had been medically refractory prior to participating in the study, any additional improvement in status was positive though evidence of efficacy was not as strong as a direct head-to-head comparison to a sham condition.

The participants completed the same questionnaires that have been used in all of our prior neuromodulation trials, namely the DHI, MBRS, and the HADS depression and anxiety subscales. The numbers of participants were too small to determine the main parameters that drove efficacy but four participants chose anti-phase and eight participants chose in-phase stimulation. There was no difference in mean efficacy between the anti-phase and in-phase treatments because of the very large degree of variance in responses. We had previously shown that the anti-phase condition was usually more effective than the in-phase condition in reducing synchrony as measured by the auditory steady state response (19). The in-phase condition in some participants was more effective, however. In some individuals, the slight phase delay in fronto-occipital transmission might be sufficient to render in-phase stimulation to be desychronizing. There were too few instances of each response category to determine which outcome measure had the strongest correlation with overall perception of treatment response but there was general aggregation of responses correlating with the DHI (Table 3A) followed by the MBRS (Table 3B) and less so with the HADS (Tables 3C,D). It should be noted that not all symptoms of MdDS were adequately captured by these scales. For example, one participant reported that her brain fog had resolved after the treatment despite persistence of her rocking vertigo.

Unfortunately, the study timeline was fixed so it was ended before more participants could try additional protocols. It is possible that some participants may have had a better ultimate response if given more opportunities to tailor their treatment. Correctly attributing treatment efficacy may also be difficult because of constant sources of motion in daily life and other changes such as home and job relocations, dietary and exercise changes, and medication changes for reasons other than MdDS.

Though home-based tACS has not been as prevalently tried as tDCS, new studies are emerging; treatment of MdDS with tACS is currently one of the few reported. A double-blind randomized sham-controlled trial of low intensity tACS (0.4 mA) at 140 Hz over visual cortex was used for migraine abortive treatment in 25 participants (16 real, 9 sham). Stimulation was provided with the NeuroConn, which could store stimulation sessions though not reporting in real-time. Participants had a mean age of about 30 years and had experienced a mean of 14 years of migraines. Adherence was low (25 of 40 completers) but the percentage of aborted migraines at 2-h in the treatment group (14 of 38 migraines, or 37%) was significantly higher than in the sham group (0 of 23 migraines, or 0%) (32). Despite the high efficacy, the main driver of low adherence was the difficulty in setting up the stimulation to treat acute migraines.

Additionally, a recent pilot study reported on two 79-year old patients with Alzheimer's related dementia in which focal 40 Hz stimulation was given over the left angular gyrus using the Starstim Neuroelectrics simulator. The 70 sessions performed over 14-weeks were administered by a spouse with excellent tolerability and adherence. Improvement was assessed on the non-visual Montreal Cognitive Assessment (25). Though current shunting through the cerebrospinal fluid can present a problem when electrodes are placed at a distance on the scalp, focal stimulation using a 4 × 1 montage with placement informed by electrical field modeling as in this study may be able to address shunting issues.

### Limitations and Challenges

The study used an open label design in order to optimize study management issues. Though we did not have a sham condition in this study, future remotely-monitored NIBS studies could allow investigators to remotely change stimulation settings in a blinded manner. A remote study would remove the ethical dilemma of requiring participants to travel to a study site and potentially receive sham stimulation in the setting of raising the risk of travel related symptom exacerbation. These studies could be done in a triple blinded manner in which the participant, the principal investigator, and the data analyst were all blind to the treatment allocation.

We note that all of our participants had participated in a more intensive on-site study and thus understood what the experience of tACS would be like. They were already adept at using on-line diaries. They had been sent their own stimulation cap which had been measured for them. In future iterations of the study, participants could be walked through how to make head circumference measurements themselves in order to snap the electrodes into the correct place.

Though most of these components were designed to be user-friendly for individuals who can casually use a computer, this may not be the case for all potential participants. Making accurate assessments of participant comfort and capability are critical to making remotely-monitored NIBS a sustainable treatment option with some individuals potentially needing the help of a second operator for set-up and maintenance.

### Future Use of Non-invasive Brain Stimulation

Remotely-monitored NIBS can increase the number and type of patient who can access research studies and ultimately clinical care using these therapies. Specifically, patients who live in rural areas far away from major research centers, those with jobs with inflexible hours, families with childcare obligations, and patients with limited transportation options would be the most likely to benefit. The protocols for this MdDS study were developed through determining functional connectivity markers that correlated with symptom improvement in MdDS. These tools could be adapted to study other functional neurotological disorders to develop diagnosis or even symptom specific protocols.

Navigating safety, feasibility, and user feedback for NIBS methods may allow a future in which NIBS is prescribed just as patients are currently prescribed medications. Patients are currently entrusted to manage their own medications with incredible freedom. Education and proper respect for the limitations of what NIBS can achieve for treating neurotological disorders are needed. As with medications, patients should be advised that more is not always better, that treatment effects may not only plateau but potentially worsen with more sessions, and that protocols that are prescribed for one individual should not be used on other people.

If NIBS could be provided with device protections such as capping the total amount of current deposited in a treatment session, aborting sessions that have high impedances, and restricting the number of sessions that could be performed per month, there may be enough built-in protections to allow patients to be given autonomy in treating themselves. They may even develop a sense of self-efficacy from managing their own treatment. Just as some medications are best taken in the morning, others at night, and many require multiple doses throughout the day, this will likely be true of NIBS treatments. Since it is impractical for treatment providers to monitor every session of NIBS in real-time, stimulation devices may be configured to send usage reports, build in side effect reporting, and send urgent notifications for more serious side effects. Patients do require different intensities of supervision as well as the need for live interactions with either the research staff or care provider in order to maintain a therapeutic relationship. On-going user feedback is important in titrating an optimal amount of supervision that balances safety, autonomy, adherence, and efficacy.

## Data Availability Statement

The datasets presented in this article are not readily available because an appropriate data sharing agreement must first be agreed between institutions before they can be shared. Requests to access the datasets should be directed to ycha@umn.edu.

## Ethics Statement

The studies involving human participants were reviewed and approved by Western IRB. The patients/participants provided their written informed consent to participate in this study.

## Author Contributions

Y-HC designed the concept of the study, performed recruitment, and wrote the manuscript. JR performed data analysis and reviewed the manuscript. DG performed recruitment, data management, and participant management. BD performed data management, remote monitoring, and technical troubleshooting. All authors were involved in some combination of design, data collection, analysis, and manuscript preparation.

## Funding

This work was supported by the Laureate Institute for Brain Research, the William K. Warren Foundation, the MdDS Balance Disorders Foundation, the Springbank Foundation, NSF EPSCoR RII Track-2 #1539068, and NIH/NIGMS grant P20 GM121312.

## Conflict of Interest

The reviewer CC declared a past co-authorship with one of the authors Y-HC to the handling Editor. The remaining authors declare that the research was conducted in the absence of any commercial or financial relationships that could be construed as a potential conflict of interest.

## Publisher's Note

All claims expressed in this article are solely those of the authors and do not necessarily represent those of their affiliated organizations, or those of the publisher, the editors and the reviewers. Any product that may be evaluated in this article, or claim that may be made by its manufacturer, is not guaranteed or endorsed by the publisher.
