# Supplementary material for: Remotely Monitored Home-Based Neuromodulation With Transcranial Alternating Current Stimulation (tACS) for Mal de Débarquement Syndrome
Source: Front Neurol. 2021 Dec 9;12:755645. doi: 10.3389/fneur.2021.755645 (PMC8695966; doi:10.3389/fneur.2021.755645)
Supplement: Supplementary file 1 [file Table_1.docx]

**Appendix:**

**MdDS Balance Rating Scale**

1. No rocking.
2. Barely noticeable rocking. Walking is normal.
3. Mild intermittent rocking that can be easily ignored. Walking is normal.
4. Mild persistent rocking, which is distracting.
5. Moderate intermittent rocking which requires extra attention to balance control. Walking is normal.
6. Moderate persistent rocking that leads to balance difficulty. May occasionally stumble.
7. Moderately severe, but intermittent rocking that impairs walking. Need occasional assistance to walk.
8. Moderately severe persistent rocking that leads to great balance difficulty. Need assistance to walk for greater distances or specific situations.
9. Severe rocking causing great difficulty with walking. Need constant gait assistance.
10. Severe rocking that prevents any walking.
